# The well-being of newly regularized migrant workers: Determinants of their satisfaction with life as compared to undocumented migrant workers and regular local residents

**DOI:** 10.1186/s40878-021-00244-2

**Published:** 2021-08-17

**Authors:** Claudine Burton-Jeangros, Aline Duvoisin, Liala Consoli, Julien Fakhoury, Yves Jackson

**Affiliations:** 1 Institute of Sociological Research, University of Geneva, 40, bd du Pont d’Arve, 1211 Geneva 4, Switzerland; 2 Swiss NCCR “LIVES - Overcoming Vulnerability: Life Course Perspectives”, University of Geneva, Geneva, Switzerland; 3 Center for the Interdisciplinary Study of Gerontology and Vulnerability, University of Geneva, Geneva, Switzerland; 4 Division of Primary Care Medicine, Geneva University Hospital and University, Geneva, Switzerland

**Keywords:** Satisfaction with life, Migration, Regularization policy, Social and economic determinants

## Abstract

Subjective assessments of well-being are becoming routine indicators, considering that material resources are insufficient to capture people’s satisfaction with life. Examining the unique situation of undocumented migrant workers, driven by aspirations for a better life but constrained by their limited rights in the country of destination, we assess their satisfaction with life and the factors that matter in their evaluations. Data were collected in Geneva (Switzerland), in a study comparing those who have just received a residency permit or about to obtaining it after submitting a regularization request (*n* = 195) with those who were still undocumented and/or had not submitted a regularization request at the time of our study (*n* = 231). In addition, comparisons were made with a sample of regular local residents (*n* = 175). Data obtained through standardized questionnaires include a range of material and non-material determinants, some unique to migrants and others common to the three populations. Satisfaction with life is significantly lower among undocumented migrant workers while those who are regularized and regular local residents report similar levels of well-being. Social participation, self-reported health and discrimination are associated to satisfaction with life among undocumented migrant workers. Among those being regularized, having been longer in the country of destination is associated with lower well-being. Among regular local residents, the only significant factor for a better satisfaction with life is having a partner. Material determinants, while distributed in vastly different levels, do not influence satisfaction with life. Despite the high satisfaction expressed by those who have recently been regularized, policy intervention still have to pay attention to their persisting difficult socioeconomic circumstances amidst a context of overall affluence.

## Introduction

When considering standards of living conditions in a high income country like Switzerland, it is easy to consider undocumented migrant workers [UMW] – migrants without a valid residency permit in the country of destination and who have never gone through an asylum procedure – as a particularly vulnerable category. The absence of a residency permit exposes them to low wages, forbids their access to social insurance schemes and to health care. On top of that, their status oblige them to adopt a low profile in their daily activities to limit the risk of being deported. In addition, when compared to positions occupied in the country of origin, undocumented economic migration is usually associated with downward occupational mobility since work opportunities in the destination country are concentrated in the lower spectrum of occupations, typically in construction work or domestic work. Previous education degrees are not acknowledged in destination countries implying that UMW are often overqualified for the work they accomplish. From a Swiss citizen or official point of view, such living conditions are difficult to understand and certainly place UMW in liminal and fragile positions.

At the same time, income gained after migration is high compared to the standards of the country of origin. In addition, UMW come from contexts in which work relations are less formalized and they might consider acceptable to be employed in the irregular economy after migration. Their tendency to think their situation is temporary – because of either an expected return to the country of origin, or the hope of becoming regular – might further help them to cope with the encountered difficulties. Overall, aspirations for a better life typically associated with economic migration might justify efforts to overcome such precarious conditions.

These contrasted elements indicate the importance to assess well-being directly from people’s own perspective, in line with the move towards studying quality of life, i.e. a subjective appraisal of living conditions - initiated a few decades ago in research (Campbell, 1981) and more recently adopted by governmental institutions. Indeed, the unique situation of UMW, as compared to the regular resident population of both the origin and destination countries, emphasizes the importance of asking them to assess their satisfaction with life. Beyond the academic interest for non-material criteria in well-being measurement, such knowledge can inform policy measures taking into account the contrasting needs of different social groups.

The canton of Geneva in Switzerland put into place a major regularization programme in 2017–2018 (Operation Papyrus) aiming at providing an annual renewable work and residency permit to several thousands of long term UMW meeting certain strict criteria. Unlike other major cities in the country, the local government thus decided to acknowledge the presence and contribution of these workers by offering them a residency permit while adopting measures aiming at reducing the irregular economy and regulating the domestic work sector. In the context of this unique policy, we developed a quasi-experimental project aiming at assessing the impact of regularization on the socioeconomic and health conditions as well as well-being of UMW, a majority of them being women – originating from South America or the Philippines – and working in the domestic work sector (Jackson et al., 2019). Available data for Switzerland suggest that they represent the largest share of undocumented migrants, followed by migrants coming from Eastern Europe countries (outside the European Union or the European Free Trade Association), Africa and Asia (Morlok et al., 2015).

The design of the longitudinal project will allow to assess how well-being changes along the acquisition of legal rights through regularization. Nevertheless this article adopts a cross-sectional approach presenting the baseline results regarding satisfaction with life of newly regularized migrant workers who had just received an annual residency permit or were about to obtaining it after submitting their application, compared to two distinct groups living in the same environment: on one hand a group of undocumented migrant workers, on the other hand regular local residents (including Swiss citizens and migrants with long-term residency permit).

## Developments of well-being research

Over the period of exceptional affluence encountered by high income countries after the Second World War, material living conditions improved across all strata of the population. In parallel to this overall trend, social science research shifted its initial focus on negative outcomes (such as poverty or illness for example) towards positive aspects of life and thus became interested in measuring individuals’ well-being or quality of life (Bartram, 2012; Campbell, 1981; Ehrler & Bühlmann, 2016). The interest for people’s own views on their circumstances is also interpreted as a result of the overall individualization trend and more explicit claims for happiness. In other words, this shift can be associated with the declining importance of material needs once a certain affluence is reached, and growing importance of non-material needs related to relations and self-actualization (Allardt, 1976) with references to the emergence of post-materialist societies. In such contexts, once a certain level of material resources is reached, it is expected that factors affecting quality of life broaden and encompass a larger range of aspects. This perspective was initiated by economists’ observation that well-being does not increase in a linear manner with income, at both the collective and individual levels. This paradox, pointed out by Easterlin, generated vivid debates over the years, but is still widely referred to (Bartram, 2015) and interpreted along different treadmill theories considering that an increasing income modifies expectations (D’Isanto et al., 2016).

The growing awareness that dimensions of well-being defined by researchers might not correspond to the dimensions that members of the public consider important for assessing their own quality of life (Le Moigne, 2010) prompted developments of research on well-being across disciplines. Initial efforts were undertaken in economics and psychology, later followed by sociology (Bartram, 2012). In the context of healthcare, quality of life also became an important factor in the assessment of patients, acknowledging that adding years to life was an insufficient indicator of medical progress (Armstrong & Caldwell, 2004). The development of research on well-being was however not unchallenged since some consider taking into account individuals’ qualitative views on their own situation as too subjective, hence biased.

Indeed, a distinction is classically made between objective and subjective well-being. The former is defined as: “the degree that a life meets explicit standards of the good life, as assessed by an impartial outsider” (Veenhoven, 2000, p. 4), based on quantifiable indicators such as income, extent of social relationships, or access to rights. In contrast, subjective well-being is self-assessed through the perceptions of one’s own experience (Bartram, 2012). In this case, individuals’ reflexive position on their current situation is often decomposed between an affective or emotional component of well-being, or happiness related to individual personality characteristics, and a cognitive component, defined as satisfaction with life. This component connects individuals to their social contexts, considering that these are likely to shape aspirations hence contributing to differences across social groups.

In that respect, Veenhoven (2000) offers two important distinctions to tackle the complexity of quality of life. First, ‘life chances’ and ‘life results’ should be considered separately since opportunities and outcomes are not necessarily aligned: some possibilities are actually not realized while some people still reach favourable outcomes despite poor opportunities. Second, external and internal qualities differentiate between conditions pertaining to the context and those associated to individuals. The intersection of these dimensions defines four types of quality of life: 1) ‘Livability’ refers to external life chances: what opportunities are offered by the context? 2) ‘Life-ability’, or capability, refers to internal life chances: what are the capacities of the individual to cope and adapt to the environment? 3) ‘Utility of life’ relates to global judgments about the good life, the ‘playground of philosophers’ (p.14) in Veenhoven’s terms; 4) ‘Appreciation of life’ relates to individual satisfaction with life, that will be at the center of the empirical analyses presented below.

Despite debates and the lack of a consensual definition, quality of life indicators have been progressively integrated into national statistics and international surveys. First introduced in research in the 1960s (Campbell, 1981), they have recently been more widely integrated in official statistics reporting in high income countries and supported by international agencies (such as OECD: Better life, Eurostat 2016). A range of domains are combined to tackle individual well-being; they include: material resources (income, housing, jobs) and quality of life areas (health, work-life balance, education, social connections, civic engagement, environmental quality, personal security, subjective well-being). These analyses conclude that objective and subjective well-being are not systematically correlated, hence showing the importance of assessing them separately and taking into consideration non-material resources (Bartram, 2012).

In those surveys, a number of individual determinants is typically integrated in analyses of satisfaction with life, including sociodemographic characteristics, education, income, employment, family situation and health (Bartram, 2012; D’Isanto et al., 2016; Safi, 2010). Beyond individual factors, the role of contextual elements on satisfaction with life such as social capital, neighbourhood conditions (better amenities), country’s unemployment rate, inflation or economic inequality is also sometimes assessed (Bartram, 2012).

## Well-being in the context of migration

Questions about objective and subjective well-being find a particular echo in research on migration. Considering the diversity of tracks migrants might follow over the migration process and their integration in the destination country, objective measures of well-being hardly seem justifiable. Subjective assessments might thus offer a better way to capture individuals’ actual conditions after migration and their adequacy with their aspirations: in other words, it offers “a summary indicator of their experience of the objective and subjective benefits and costs of migration that truly matter to them.” (Hendriks & Bartram, 2019, p. 289). Indeed, migration is driven by aspirations for a better life, for one’s self but also possibly for one’s relatives. Connecting the present with the future, aspirations relate to ‘hopes, plans, ambitions or goals’ (Scheibelhofer, 2018) and most likely encompass more than material elements. Indeed, while it is considered that migration is prompted by economic motives, empirical results show only a weak association between increased income gained after migration and higher well-being (Bartram, 2011; Safi, 2010), confirming observations made in general population surveys.

It is therefore important to examine these aspirations in a dynamic perspective, since both actual living conditions and expectations are likely to change over the process of migration (Boccagni, 2017), raising the challenge to assess aspirations over time and notably to capture their ‘transient nature’ (Carling & Schewel, 2018, p. 950). Indeed assessments of well-being are likely to combine different temporal states, including the evaluation of the past, the experience of the present and the expectation towards the future (Durayappah, 2011). Just after migration, conditions in the destination country might not meet the initial expectations with migrants experiencing discrepancies between their aspirations at the start of their journey and their actual living conditions after migration (D’Isanto et al., 2016). Different factors should be accounted for. First the social status occupied in the country of origin is difficult to maintain in the destination country, those who held a middle-class position are confronted with the lack of recognition of their education background and language difficulties (Bartram, 2013), potentially leading to downward social mobility. Second, general living conditions in the destination country can challenge initial aspirations which “might be intensified (and then frustrated) via direct exposure to the consumption standards of wealthy societies.” (Bartram, 2013, p. 159), reflecting the mechanism by which gains in income expands aspirations. Third, contrary to the often quoted assimilation hypothesis, suggesting that satisfaction with life should increase over time and across generations, as a result of declining migration-related stress, persistent discrimination and isolation might alter the process of integration and hamper improvement in satisfaction with life (Safi, 2010). These elements emphasize the importance of adopting a biographical or life course perspective (Scheibelhofer, 2018), following individuals over time, ideally taking into consideration their aspiration before migration and once they have settled in the destination country.

Next to assessing transformations of satisfaction with life over time, comparison mechanisms also matter. Initially migrants assess their situation compared to people they left behind, those living in the country of origin; however over time, they might start comparing their own conditions with those of natives and regular migrants living in the destination country (Hendriks & Bartram, 2019). Some empirical conclusions are worth reporting here. A study on Latin America immigrants show that, prior to migration, their satisfaction with life was lower than among their counterparts in the country of origin who planned to stay, suggesting that individuals who are unhappy are more likely to migrate (Graham & Markowitz, 2011). In destination countries, migrants' satisfaction with life is also lower than among natives or regular migrants, which could reflect a shift of comparisons, this time with a higher status reference group (Bartram, 2012; D’Egidio et al., 2016; Safi, 2010). On top of that, variations in well-being assessments across cultural areas have been observed with the importance attributed to personal achievement or attainment on one hand, the well-being of others on the other hand varying across contexts (Uchida & Ogihara, 2012).

Undocumented economic migrants, particularly motivated by aspirations for a better life, encounter specific difficulties in destination countries, notably limited access to social rights restricting employment opportunities and impeding family reunification (Torres & Young, 2016). At the same time, obligations towards relatives (partner, children, parents) who remained in the country of origin are associated with important transnational affective and economic ties (Ambrosini, 2012; Fresnoza-Flot, 2009). This is particularly the case for female migrant care workers who engage in high income countries domestic work and provide important economic support to their relatives left behind (World Health Organization, 2017) while also trying to maintain their care duties in the country of origin. The ambivalence of these transnational ties has been emphasized: on the one hand, they provide social support, even if distant, and motivations for the hardships associated with undocumented work, on the other hand they generate suffering due to the distance and moral obligations towards those left behind (Horn & Fokkema, 2020). Besides these complex relationships, qualitative research has also documented the heterogeneity of UMWs’ trajectories (van Meeteren et al., 2015). These multiple elements justify approaching well-being from the migrants’ own point of view. As regard regularization, it is largely expected that it should come with a number of benefits, beyond the economic sphere. Obtaining an official status in the host society allows legitimate social membership, increases control over one’s life and facilitates the capacity to plan for the future, which are all likely to enhance well-being (Kraler, 2019; van Meeteren et al., 2015). At the same time, the promotion of private aspirations through regularization, which reduces the gap with local residents, is likely to remain in competition with obligations towards relatives in the country of origin (Courant, 2014). Our analyses contribute to empirical quantitative research on migrants’ well-being, which remains limited (Bartram, 2015), and particularly so as regards undocumented migrants (D’Egidio et al., 2016; D’Isanto et al., 2016; Kuehne et al., 2015).

More specifically, Veenhoven’s (2000) framework is useful to analyse UMWs’ well-being since it considers opportunities and outcomes at both the individual and collective levels, thus accounting for individual agency and structural constraints. UMWs, sufficiently dissatisfied with the life chances of their country of origin, modified these chances by transforming their aspirations into an actual migration project (Carling & Schewel, 2018). However, beyond their own drive for a better life, their undocumented status has limited their life chances in the destination country, where their rights have remained limited. Our study has been set with the intention to assess their life results or appreciation of life, as measured by subjective satisfaction with life at the moment they were regularized, thus when their fuller integration and agency are likely to improve their well-being, while their material conditions are still far from equivalent to those of regular residents.

This article examines newly regularized migrant workers satisfaction with life, as compared with undocumented migrant workers, not eligible for regularization or who had not applied on one hand, and regular local residents, comparable in terms of age and employment status whose data were collected in the Swiss Household Panel on the other hand. These quantitative analyses can help to better understand the complex interplay of time and comparison mechanisms in the context of migration described above, with a focus on a particularly hard-to-reach and under-researched group of workers. After describing variations in levels of well-being, we aim at assessing whether determinants of satisfaction with life are similar, or not, across these three groups. We expect these analyses to shed some more light on the relative impact of material and non-material factors on satisfaction with life, whose respective role remains debated in the literature (D’Isanto et al., 2016).

## Methodology

### Data

Two databases were used to conduct the analyses of this paper: the ‘Parchemins study’ and the Swiss Household Panel (SHP). The ‘Parchemins study’ is a prospective study aiming at assessing the impact of regularization, e.g. access to a work and residency permit, on the health and well-being of undocumented migrant workers living in Geneva (Jackson et al., 2019). The launch of a local regularization policy in early 2017, specifically targeting undocumented migrant workers and excluding asylum seekers, offered the opportunity to develop a quasi-experimental research design through the collection of longitudinal data with two groups of undocumented migrants. Those applying for a residency permit had to provide documentation proving they met the five following criteria: 1) a stay of at least 10 years in the Canton for individuals or at least 5 years for those having school-age children; 2) basic French proficiency; 3) sufficient financial resources; 4) being employed; and 5) absence of criminal record other than related to their undocumented residency status. In this study, “regularized” migrant workers include those who just received their residency permit and those whose file has been submitted, who in the Operation Papyrus context are most likely to be granted a permit considering how the application procedure is set up. “Undocumented” migrant workers include those who have not applied. All these participants were at least 18 years old and were not nationals of a European Union country or a European Free-Trade Association country. Data were collected face-to-face on the basis of a standardized questionnaire addressing different issues, such as migration trajectory, health, work and financial situation, and social participation. The empirical analysis developed in this paper uses data of the baseline sample of 464 participants collected between October 2017 and December 2018. This study was approved by the Ethics Committee of Geneva, Switzerland and all participants gave informed consent.

The SHP is an annual panel study that started in 1999. It consists of a random sample of households of regular local residents in Switzerland, who might include some migrants, questioned on their living conditions and political and social representations. In this paper we used the wave collected in 2017–2018 and focused only on respondents living in canton of Geneva (including Swiss citizens and migrants with long-term residency permit), part of the labour force, and of the same age range (i.e. 19–74 years old) than the participants of the ‘Parchemins study’. The resulting sub-sample contains 175 individuals. The two data sources are therefore comparable in terms of period, geographic location, age, and activity status in the broad sense.

### Measures

The Parchemins and SHP databases contain the same measure of subjective well-being, which is based on a general satisfaction with life question: “In general, how satisfied are you with your life, if 0 means not at all satisfied, and 10 means completely satisfied?”. To assess the role of comparison mechanisms, we also asked participants to compare their satisfaction with life with the one of people in their country of origin and the one of local residents (better, same, worse).

Then we modeled the associations between satisfaction with life and a set of determinants of well-being based on the literature (Bartram, 2012; D’Isanto et al., 2016; Hendriks & Bartram, 2019; Safi, 2010). We included sociodemographic variables such as gender, age, and level of education. Previous research is inconclusive as regard differences between men and women, but there is some agreement about a u-shaped distribution of satisfaction with life along age (D’Isanto et al., 2016). However, our samples consist of respondents aged 18 and over, most of which are middle aged. After testing for a quadratic relationship was inconclusive, we included age linearly in our models. Education gained in the country of origin is likely to have a low return once migrants settle down in countries with higher education levels on average (D’Isanto et al., 2016). However we tested its role for satisfaction with life, with three levels of education (primary, secondary, and tertiary).

Considering the limited association between income and satisfaction with life (Hendriks & Bartram, 2019), we included 3 variables to assess the role of material resources. Individuals employment situation was assessed in a binary measure (employed, unemployed). With regard to income, we used net yearly household income equivalised, according to the modified OECD equivalence scale. We also estimated housing conditions by computing a continuous variable of the number of persons per room in the accommodation.

We assessed different dimensions of social integration. The role of the family situation was measured with two variables: being in a couple (yes, no) and having children (yes, no), these including children of all ages, thus some being minors and others being adults. UMW’s transnational family circumstances often change over time with for example partners or children initially left behind joining UMWs in the country of destination. Due to the difficulty to assess the complexity of these trajectories, we here only considered the existing relations, to assess their potential role as positive social support resources or negative sources of stress for well-being (Horn & Fokkema, 2020), but not taking into account joint residency. With regards to social participation, we included a binary variable of participation in a club or an association (yes, no).

The health dimension was assessed including a general self-rated health measure. Participants had to rate their health on a 5-points scale, which we dichotomized (“excellent”, “very good” versus “good”, “fair” “poor”). We considered that individuals reporting less than “very good” health expressed some reserves about the assessment of their own health.

Finally, we assessed 4 variables specific to migration. Duration of stay in Geneva being a criteria to apply for regularization, it is likely to be different across the two groups of migrants; however we included it as a continuous variable as a factor that could reflect transformation of aspirations over time (Hendriks & Bartram, 2019). Considering their fairly high frequency, having returned to the country of origin since arriving in Switzerland (yes, no) and sending remittances to family members (yes, no) were included to evaluate the role of maintained ties with the country of origin. We also asked participants whether they had been exposed to discrimination because of their nationality, ethnic origin or color over the last year in a number of settings such as their workplace, healthcare environments, etc. (yes, no).

### Statistical analyses

First, we compare demographic, economic, social, health and migration-related characteristics of the three groups. Categorical variables are compared using the chi-square test and continuous variables are compared using the Kruskall-Wallis test. Second, we provide descriptive analyses of the distribution of satisfaction with life across the three groups, including among migrant workers comparisons with population in the country of origin and with the Swiss population. Pairwise comparisons based on the Kruskall-Wallis test were also conducted. The observed variations justify to run stratified analyses of satisfaction with life determinants in a third step.

Multivariate analyses were conducted in two parts. In a first part, we focused on comparing factors associated with well-being across regularized and undocumented migrant workers, including some variables specific to migration (duration of stay in Geneva, return to the country of origin, remittances and discrimination). We used ordinary least squares (OLS) regressions to estimate the relationship between the continuous measure of satisfaction with life and the set of independent variables. In a second part, comparisons integrated the regular local residents sample, with fitted models including only comparable variables in the two databases. We reported the standardized coefficients of the OLS models. In this way, we could determine which independent variables have greater association with subjective well-being, not only within each model, but also between models.

## Results

### Description of the samples

Because of missing values, we included 426 of the 464 ‘Parchemins study’ participants. Among them, 54.2% are undocumented and 45.8% are regularized (Table 1, first and second columns). Three quarters of the ‘Parchemins study’ participants are women, with an average age of about 44 years. Three quarters of the sample have at least reached a secondary education level. In both groups, around half live in couple and almost two thirds have children, either living with them in Switzerland or in the country of origin. Two thirds of the sample are sending remittances to family members living in the country of origin.

**Table 1:** Demographic, economic, social, health and migration-related characteristics of the tree samples

	Undocumented *N* = 231	Regularized *N* = 195	Regular local residents *N* = 175	*p*-value
	n(%) or mean (SD)	n(%) or mean (SD)	n(%) or mean (SD)	
Women	170 (73.6)	142 (72.8)	94 (53.7)	< 0.001
Age	43.0 (10.2)	45.5 (9.9)	46.9 (10.6)	< 0.001
Education				< 0.001
Primary	51 (22.1)	43 (22.1)	9 (5.1)	
Secondary	123 (53.2)	108 (55.4)	57 (32.6)	
Tertiary	57 (24.7)	44 (22.6)	109 (62.3)	
Employed	204 (88.3)	189 (96.9)	156 (89.1)	0.003
Yearly household income equivalised, OECD, net (CHF)	23′112 (14′261)	33′789 (12′669)	83′199 (38′986)	< 0.001
Number of persons per room in the accommodation	1.9 (1.1)	1.6 (0.9)	0.8 (0.4)	< 0.001
In couple	102 (44.2)	102 (52.3)	147 (84)	< 0.001
Have children	148 (64.1)	130 (66.7)	132 (75.4)	0.044
Participation in association, club	165 (71.4)	156 (80.0)	60 (34.3)	< 0.001
Self-reported health (excellent and very good)	65 (28.1)	88 (45.3)	152 (86.9)	< 0.001
Years in Geneva	9.9 (5.0)	13.9 (4.7)	–	< 0.001
Already returned to the country of origin	99 (42.9)	125 (64.1)	–	< 0.001
Sending remittances	157 (68.0)	132 (67.7)	–	0.952
Experienced discrimination because of the origin	89 (38.5)	50 (25.6)	–	0.005

Data sources: Parchemins study and Swiss Household Panel (SHP)

On other characteristics, these two groups are however quite distinct. Regularized migrant workers have been in Geneva for a longer time (almost 14 years on average) than those who are undocumented (10 years). The former are also more often employed (97%) than the latter (88%) and have a substantially higher income (about one third). Regularized migrant workers have better housing conditions with a lower density index (1.56 versus 1.89), a higher social participation rate (80% versus 71%), and report better health (45% consider being in very good or excellent health) than undocumented migrant workers (28%). Finally, the former have returned more frequently to their country of origin (64% versus 43%) and report less discrimination (26% versus 39%) than those who are undocumented. Overall these differences could either reflect an improvement of the living circumstances over the process leading to the acquisition of a residency permit, or result from a selection induced by the regularization procedure itself.

The comparable sample of regular local residents surveyed in the Swiss Household Panel (*n* = 175) (Table 1, third column) is more balanced in terms of gender and slightly older. They have higher education attainments, with 62% having reached the tertiary level. While nine out of ten are employed (i.e. a proportion similar to the one observed in the undocumented group), their average income is three times higher than the average income of undocumented migrant workers (83′200 CHF versus 23′100 CHF). The regular local residents more often live in a couple or have children than both the undocumented and regularized migrant workers. These differences might result from the restrictions that being undocumented represent for building a family life. Finally, while regular local residents report better housing conditions and health, their participation to social activities is much lower than among the two groups of migrant workers.

### Distribution of satisfaction with life

**Fig. 1:**
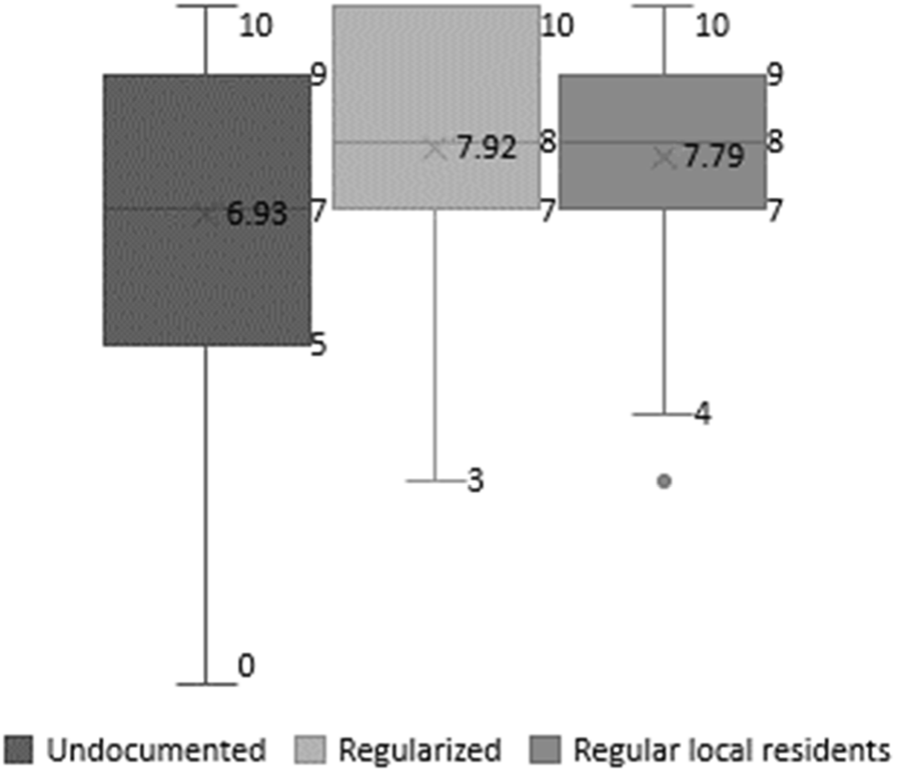
Satisfaction with life by groups. Data sources: Parchemins study and Swiss Household Panel (SHP)

Mean satisfaction with life ranges between 6.93 (out of a scale 0 to 10) and 7.92, indicating overall fairly high levels of well-being (Fig. 1). Undocumented migrant workers present the lowest value (6.93), followed by the regular local residents (7.79) and regularized migrant workers (7.92), with these last two groups not differing significantly. The highest score of the regularized migrant workers is associated with the largest number of maximum scores (10). The dispersion of answers is highest among the undocumented migrant workers, followed by the regularized migrant workers while more homogenous responses are found among the regular local residents (Fig. 1).

**Fig. 2:**
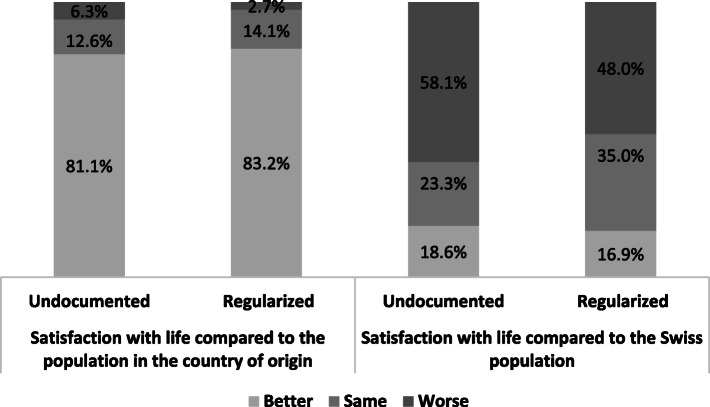
Satisfaction with life compared to the population in the country of origin and to the Swiss population by groups. Data sources: Parchemins study

When asked to compare their satisfaction with life with the one of others, more than 80% of undocumented and regularized migrant workers estimated that theirs was better than the one of people living in their country of origin (Fig. 2). However, significant differences emerged in the comparison with the Swiss population: regularized migrant workers evaluated more frequently their satisfaction with life as identical to the Swiss population than the undocumented migrant workers (35% versus 23%), and the latter more often rated their satisfaction with life as worse than the Swiss population (48% versus 38%). These results could reflect either an evolution of aspirations among regularized migrant workers, or their improved social circumstances.

### Determinants of satisfaction with life

First, we compare the determinants among the two groups of migrant workers, having shown above that beyond their legal status, they are quite distinct as material resources and social integration are concerned (Table 2). Among those who are undocumented, age, gender and education do not make any significant difference in their level of satisfaction with life. Factors associated with a higher satisfaction level include: a higher social participation and a better self-reported health. A contrario, housing conditions, being employed, the amount of material resources, as well as the family situation (being in a couple or having children) are not associated with satisfaction with life. With regards to specific migration factors, the time spent in Geneva and ties with the country of origin (have returned to the country of origin and sending remittances to family members) are not associated with satisfaction with life. However, the experience of discrimination significantly lowers their level of well-being.

Among regularized migrant workers, age, gender and education have no significant impact on satisfaction with life. In this group either, material resources, family circumstances and health do not significantly matter for satisfaction with life. The only significant factor is the time spent in Geneva: a longer stay is related to lower satisfaction with life.

A comparative analysis of these determinants shows that sociodemographic characteristics, education, income and housing density, as well as family circumstances are not related to satisfaction with life in either group of migrant workers. The low or absent effect of income already observed in the literature (D’Isanto et al., 2016; Hendriks & Bartram, 2019) is therefore confirmed in our study.

The fact that, among regularized migrant workers, the length of stay in Geneva is negatively associated with satisfaction could reflect growing frustration over time, considering that achievements in the country of destination are not up to their initial expectations. It could also be due to their stronger commitment towards the country of destination, as made concrete by their application for regularization, and associated with a shift in comparisons moving from the country of origin towards the country of destination.

Good health status matters for both groups, only in a limited manner for the regularized migrant workers but significantly so for those who are undocumented (standardized coefficients = 0.13 versus 0.25). The rather poor health status of undocumented migrants is thus of great relevance for them. Indeed, since being an undocumented economic migrant puts a lot of emphasis on the capacity to work, as an unconditional condition to stay in the country of destination, poor health can seriously jeopardize this objective, hence affecting well-being. Their limited access to healthcare as a result of their lower health insurance coverage is an additional source of preoccupation likely to affect their satisfaction with life.

Social participation also appears important for satisfaction with life. While it is not significant for regularized migrant workers, it is so for those who are undocumented. The instrumental and emotional support associated with social participation might be particularly important for those who have no legal rights and weaker social connections (as observed by their lower family engagements than regular local residents). The negative impact of self-reported discrimination among undocumented migrant workers is particularly noteworthy, as compared to the regularized migrant workers who experience it less and whose satisfaction with life is not impacted by it.

**Table 2:** Determinants of satisfaction with life across undocumented and regularized migrant workers

	Undocumented		Regularized	
	Standardized coefficients	*p*-value	Standardized coefficients	*p*-value
Woman (ref. Male)	0.10	0.11	0.07	0.35
Age (year)	0.11	0.16	0.07	0.40
Education (ref. Secondary)				
Primary	− 0.01	0.86	0.12	0.13
Tertiary	−0.01	0.83	−0.01	0.92
Employed (ref. Unemployed)	0.07	0.32	−0.11	0.17
Yearly household income equivalised, OECD, net (in thousands CHF)	0.10	0.15	0.08	0.35
Number of persons per room	−0.05	0.41	−0.14	0.09
Partner (ref. No)	0.08	0.22	0.04	0.64
Children (ref. No)	0.02	0.77	0.07	0.36
Participation in association, club (ref. No)	0.18	0.00	0.13	0.08
Self-rated health (ref. Poor to good)	0.25	0.00	0.13	0.08
Years in Geneva	−0.01	0.85	−0.17	0.04
Return to the country of origin (ref. No)	0.10	0.12	0.07	0.35
Remittances (ref. No)	0.12	0.07	0.10	0.20
Discrimination (ref. No)	−0.13	0.03	0.06	0.42

Data source: Parchemins study

The second step of analysis includes the regular local residents (Table 3). Among migrant workers, gender, employment status, housing conditions, and being a parent do not affect the satisfaction with life in any of the two groups. Income is associated with satisfaction with life for undocumented migrant workers but as we have seen before (Table 2), this association is no longer significant when migration factors are taken into account. More precisely, the inclusion of sending remittances removes the significant effect of income in this group. This result suggests that it is not the level of income per se that has an influence on UMW satisfaction, but having enough income to send remittances to family members. For regularized migrant workers and regular local residents, income does not matter.

**Table 3:** Determinants of satisfaction with life across migrant workers and regular local residents

	Undocumented		Regularized		Regular local residents	
	Standardized coefficients	*p*-value	Standardized coefficients	*p*-value	Standardized coefficients	*p*-value
Woman (ref. Male)	0.12	0.06	0.10	0.23	0.09	0.26
Age (year)	0.14	0.04	−0.02	0.81	0.02	0.84
Education (ref. Secondary)						
Primary	−0.03	0.68	0.12	0.10	−0.08	0.29
Tertiary	−0.06	0.39	0.00	1.00	−0.17	0.05
Employed (ref. Unemployed)	0.09	0.19	−0.12	0.13	0.05	0.50
Yearly household income equivalised, OECD, net (in thousands CHF)	0.14	0.04	0.11	0.21	0.16	0.08
Number of persons per room	−0.05	0.39	−0.14	0.10	−0.11	0.22
Partner (ref. No)	0.08	0.24	0.02	0.85	0.23	0.00
Children (ref. No)	0.04	0.55	0.12	0.11	−0.11	0.21
Participation in association, club (ref. No)	0.18	0.00	0.12	0.10	0.04	0.62
Self-rated health (ref. Poor to good)	0.26	0.00	0.14	0.08	0.14	0.06

Data sources: Parchemins study and Swiss Household Panel (SHP)

Self-reported health remains significant for undocumented migrant workers but has no significant impact for regularized migrant workers and for regular local residents. Social participation follows a gradient: it is positive and significant for undocumented migrant workers (0.18), positive for regularized migrant workers but not significant (0.12), while it shows no association at all with satisfaction with life among regular local residents. For them, the only significant factor affecting their satisfaction with life is having a partner, which does not matter in both groups of migrant workers.

## Discussion and conclusion

Considering the recent interest for satisfaction with life as a subjective measure encompassing individuals’ own assessments for their current circumstances, we have compared three groups having starkly different living conditions. We consider the ability to compare quantitative data across undocumented, regularized migrant workers and regular local residents a strength of this study. Participants to the ‘Parchemins study’ present socio-demographic characteristics fairly close to the available descriptions of undocumented migrant workers in Switzerland (Morlok et al., 2015) and in canton of Geneva (Flückiger et al., 2012).

The lowest satisfaction with life of undocumented migrant workers was expected, reflecting their particularly difficult living conditions, including limited rights but also the uncertainty they face regarding the future. The fact that newly regularized migrant workers reported as high satisfaction with life as regular local residents confirms the benefits associated with obtaining an official status (Kraler, 2019). This high satisfaction with life of regularized migrant workers could reflect the recent alleviation of a long-term stress associated with their undocumented status, getting a work permit representing an official acknowledgment of their presence in Geneva and of their contribution to the local economy. Becoming regular residents, a turning point in their migratory trajectory, is indeed putting an end to years of struggle through which these migrants managed to stay. Their high level of satisfaction with life could also reflect the benefits expected to come with the regularization, including access to the regular labour market, possibly a better acknowledgment of past education and more possibilities to travel back and forth with the country of origin. With increased control over one’s life, regularization is likely to expand their aspirations, a process potentially strengthened by the fairly strict selection process of Operation Papyrus which could have reinforced their feeling of deservingness (Chauvin & Garcés-Mascareñas, 2014).

If becoming regular certainly modifies their life chances (Veenhoven, 2000), their actualization is however likely to encounter some obstacles. Indeed becoming regular is associated with new material and non-material preoccupations, including paying taxes and mandatory health insurance as well as meeting conditions to renew a first residency permit which was limited in time.

Findings on comparisons with others suggest first that, independently of their current situation, a large majority of migrant workers consider their quality of life higher than in the country of origin, an assessment justifying the multiple difficulties they struggled with. The fact that once they are regularized, more consider reaching similar levels of well-being as the general population of the host country could reflect their feeling of being progressively integrated and not so much different from the rest of the population anymore, despite vastly different material conditions across the two groups.

Indeed, material factors only marginally affect satisfaction with life in any of the groups, as observed in other studies including among undocumented migrants (D’Isanto et al., 2016). This certainly confirms the importance to not assess quality of life along economic resources or material factors only. The high level of remittances across both groups of migrant workers shows the persistence of obligations over time. For those who are not documented this burden seems more specifically associated with the level of income: as shown in Table 2, what matters for them is not so much their income but the fact that it allows them to send money at home regularly, thus responding to the expectations associated with their migration.

Among other migration-related factors, the negative role of experiences of discrimination observed among those who are undocumented emphasizes their most marginal and fragile position in the country of destination, even after several years. It also suggests that those who have no legal rights and consequently fear deportation are more sensitive to these experiences of discrimination. The fact that this determinant is not relevant for those who are regularized speaks for the importance of their progressive integration and legitimate position in the host society. For the latter, what matters is the length of time spent in Geneva: the negative effect of a longer stay on satisfaction with life might express their frustrated aspirations, at the intersection of their original goals when they migrated, their actual situation years later and their possibly new aspirations for the future, once progressively integrated in the country of destination. It might also reflect an exacerbated ambivalence as regard plans for the future, the possibility to stay in Switzerland conflicting with the maintenance of transnational ties and/or previous intentions to go back to the country of origin.

Other non-material determinants matter differently across the groups. For undocumented migrant workers, the significant importance of health reflects the fact that the capacity to work is incompressible; while not significant for the others, this factor is still important in their evaluation of satisfaction with life, as previously observed in the literature (D’Isanto et al., 2016; Safi, 2010). The positive role of social participation for those who are undocumented is another signal of their marginal position in the country of destination, suggesting the importance of support offered by interactions with others (van Meeteren et al., 2009). Finally, family circumstances do not affect satisfaction with life of those who have migrated, which does not permit to confirm that family ties are either resources or stressors (Horn & Fokkema, 2020); our data thus do not allow to account for the heterogeneity of family residence trajectories and their ambivalent effects on well-being. The fact that having a partner is the only significant determinant of satisfaction with life of regular local residents might reflect the importance gained by individualistic and expressive preoccupations.

These findings do not come without limitations. The sample of undocumented and regularized migrant workers, which excludes asylum seekers, is purposive and thus likely to represent only a portion of undocumented migrants, i.e. those who are more connected with associations and institutions through which we recruited them. Furthermore, the large share of female migrants originating from South America and working in the domestic sector makes it difficult to assess the influence of the country of origin and the labor sector on well-being. The sample is however quite unique in a field where quantitative research is still limited (D’Isanto et al., 2016). To better understand the role of contextual and biographical factors, satisfaction with life should also be measured before migration, in studies initiated prior to departure, such as the Health of Philippine Emigrants Study (HoPES) (Morey et al., 2020).

In conclusion, satisfaction with life reflects subjective appraisals of the adequacy between aspirations and living circumstances. Both aspirations and living conditions depend on opportunities provided by different contexts. People who migrate not only compare places but actually put their ambitions for a better life into practice through the decision to leave (Carling & Schewel, 2018). However their capacity to meet their expectations will be constrained by conditions available to them in countries of destination. Our study shows that the possibility to obtain a residency permit is favorable for well-being. Indeed those newly regularized rated their satisfaction with life as high as regular local residents, while the latter benefit from much better economic circumstances. As emphasized by the literature and our results, what matters for well-being varies along both local standards and individual expectations. Therefore, following the turning point of becoming an official member of the host society, comparisons and aspirations are likely to shift progressively. The positive assessment of those who just gained legal rights should not serve to hide the overall persistent and difficult socioeconomic circumstances of these migrant workers occupying crucial but little valued positions, notably in the domestic care sector (World Health Organization, 2017). Our findings thus call for policy interventions addressing the changing needs of those remaining vulnerable amidst a context of overall affluence.

## Data Availability

Two datasets are used: - The main data was collected in the context of the Parchemins study (Jackson et al., 2019). This data is currently not publicly available, but as specified in the Data Management of the Study will be deposited in an open archive after the project is completed. They are available from the corresponding author on reasonable request - This study has also used data collected by the Swiss Household Panel (SHP), which is based at the Swiss Centre of Expertise in the Social Sciences FORS. The project is financed by the Swiss National Science Foundation. This data is publicly available 10.23662/FORS-DS-932-2.
